# Cross-talk between HIF and PD-1/PD-L1 pathways in carcinogenesis and therapy

**DOI:** 10.1172/JCI159473

**Published:** 2022-05-02

**Authors:** Michael R. Shurin, Viktor Umansky

**Affiliations:** 1 Departments of Pathology and Immunology, University of Pittsburgh Medical Center, Pittsburgh, Pennsylvania, USA.; 2 Skin Cancer Unit, German Cancer Research Center (DKFZ), Heidelberg, Germany.; 3 Department of Dermatology, Venereology and Allergology, University Medical Center Mannheim, Ruprecht-Karl University of Heidelberg, Mannheim, Germany.

## Abstract

Tumor-associated hypoxia plays an important role in carcinogenesis and metastasis. The expression, activation, and stabilization of hypoxia-inducible transcription factors (HIFs) support malignant cell survival, proliferation, plasticity, and motility. Hypoxia also upregulates the expression of programmed cell death ligand 1 (PD-L1) in malignant and immune regulatory cells. Therefore, the combination of HIF inhibitors and checkpoint inhibitors (CPIs) is promising for boosting antitumor immunity and diminishing malignant cell plasticity and therapy resistance. In this issue of the *JCI*, Salman et al. report the development of a specific agent that inhibited HIF-1/2–mediated gene expression in tumor cells and suppressed tumor growth. Bailey, Liu, et al. in this issue demonstrate that targeting HIF-1**α** abrogated PD-L1–mediated immune evasion by suppressing PD-L1 expression on malignant and myeloid regulatory cells, causing tumor rejection. These findings suggest that targeting the HIF/PD-L1 axis with specific HIF inhibitors should improve the safety and efficacy of CPIs for cancer therapy.

## Hypoxia-inducible factors in cancer

Hypoxia, which is also known as oxygen deficiency and is commonly seen in the solid tumor environment because of an erratic tumor vasculature and high metabolic rate, plays an important regulatory role in cancer development, tumor growth and spreading, formation of distant metastases, and cancer dormancy. The essential molecular issue in hypoxia is the activation and stabilization of hypoxia-inducible transcription factors (HIFs) (1). HIF-1, the main transcription factor activated in hypoxic conditions, is composed of two subunits, α and β, which have similar conformations. Under hypoxic conditions, when HIF-1α is stable, it translocates to the cell nucleus and heterodimerizes with HIF-1β. In the nucleus, HIF-1 initiates the transcription of numerous genes regulating cellular energy metabolism, function, and survival, and are collectively known as hypoxia-responsive genes (1). There are also two homologs that associate with HIF-1α; HIF-2α shares some functionality and targeted genes with HIF-1α, and HIF-3α may negatively regulate HIF-1α and HIF-2α functions (2).

**Figure 1:**
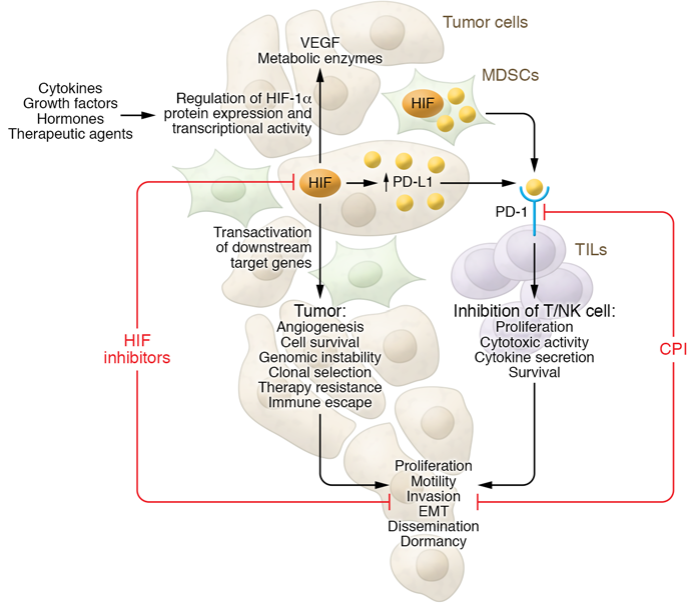
Cross-talk between HIF and PD-1/PD-L1 pathways in carcinogenesis and therapy. A majority of solid tumors develop hypoxia because of chaotic vascularization leading to deprivation of optimal oxygen supply and increased cellular proliferation and metabolic rate. Hypoxia causes the activation and stabilization of key transcription factors, the hypoxia-inducible transcription factors (HIFs). HIF-1α and HIF-2α control the expression of many tumorigenesis genes. HIFs promote tumor progression by stimulating angiogenesis, immunosuppression, EMT, and metabolic reprogramming, and by supporting malignant cell survival, motility, proliferation, plasticity, and enhancing treatment resistance and escape from a nutrient-deprived milieu. Hypoxia also upregulates the expression of PD-L1 in malignant cells and MDSCs via HIF-1α and HIF-2α. PD-L1, via binding to its receptor (PD-1), acts as a protumorigenic factor that induces immune tolerance within the tumor microenvironment and inhibits antitumor immune responses by suppressing activity of tumor-specific TILs. HIF inhibition synergizes with CPIs to block tumor development and progression. Further studies should determine how other factors in the tumor milieu, such as cytokines, chemokines, growth factors, hormones, and therapeutic agents, may interfere with the antitumor potential of a combination of HIF inhibitors and CPI therapy.

HIF-1 promotes tumor progression via the stimulation of angiogenesis, immunosuppression, epithelial-mesenchymal transition (EMT), and metabolic reprogramming, supporting malignant cell survival, proliferation, plasticity, and motility (ref. 1 and Figure 1). Transient acute hypoxic conditions and chronic hypoxia generate a metabolically diverse tumor microenvironment (3), which induces an intense conditional pressure on cancerous cells that encourages the survival of aggressive malignant clones. Hence, in a clinical setting, hypoxia correlates with worse prognosis in different cancer types and, in addition, imparts resistance to different therapeutic approaches (4).

Numerous experimental and clinical data reveal that hypoxia induces radioresistance and resistance to a variety of chemotherapeutic agents, affects antitumoral immune responses, and controls crucial regulatory pathways, impacting key aspects of cancer biology. For instance, hypoxia impacts the efficiency of cancer immunotherapy by contributing to the immune-eliminated phenotype, which is a key barrier to the therapeutic effect of adoptive cell therapy (5). Therefore, hypoxia signaling in cancer is an apparent target for intervention and the suppression of hypoxia-associated signaling may be considered a valuable treatment option in diseases with limited therapeutic choices. Clinical studies of HIF inhibitors indicate benefit and justify further evaluation either as a single agent or in combination with other antitumor agents (6).

## Targeting HIFs

Various signaling pathways control the expression, accumulation, and activity of HIF-1 protein throughout its signaling cycle inside cells, suggesting that targeting HIF-1 might be an encouraging approach for cancer drug development. In fact, several approaches that target hypoxic malignant cells are presently in preclinical and clinical evaluation. Many small molecules that target HIF-1α at the mRNA or protein level block HIF-1α and HIF-1β heterodimerization, promote HIF-1 degradation, or interfere with DNA-binding activity in the nucleus to suppress HIF-1 activity have been introduced in recent years (7). For instance, two small molecule HIF-2α inhibitors, MD6482 (also known as PT2977) and PT2385, exhibited promising activities in experimental models, and based on the results of clinical testing in participants with advanced renal cell carcinoma, MK6482 became the first FDA-approved HIF-2α inhibitor (1). Antisense oligonucleotide or shRNA can downregulate HIF-1/2α (8). Similarly, agents can block HIF protein synthesis or decrease HIF stability (9, 10). PX-478, a melphalan derivative, lowers HIF-1α levels by inhibiting HIF-1α deubiquitination, decreasing HIF-1α mRNA expression, and reducing HIF-1α translation (11). Several chemotherapeutic agents in clinical use might affect HIF-1α activity. For example, rapamycin can inhibit hypoxia-induced HIF-1α expression (4), while doxorubicin and daunorubicin inhibit HIF-1 by blocking its binding to DNA (12).

Other data show that HIF-1 inhibitors may contribute to the antiangiogenic and antitumor effects of different agents that target signal transduction pathways. For instance, the DNA alkylating agent temozolomide demonstrated greater antitumor efficacy if applied together with HIF-1 inhibition in a glioma model (13). Alternatively, some approved drugs known to indirectly alter the HIF-1α pathway could also act as adjuvant therapy for existing cancer treatments (6). For example, mTOR inhibitors, known to translationally attenuate HIF-1α protein expression, synergize with rapamycin in reducing tumor growth in preclinical models of hepatocellular carcinoma (14).

The combination of HIF-1 inhibitors and checkpoint inhibitors (CPIs) may provide a particularly promising treatment; the CPIs would support antitumor immunity and the HIF-1 inhibitors would counteract the capacity of malignant cells to adapt to the subsequent conditions and develop therapy resistance (Figure 1). This suggestion is based on data showing that a hypoxic environment permits escape of cancerous cells from killing by cytotoxic T lymphocytes (CTLs) and natural killer (NK) cells via granzyme B degradation and TGF-β–containing extracellular vesicles (15, 16). In addition, hypoxia upregulates expression of immune checkpoint molecules, such as VISTA and programmed cell death ligand 1 (PD-L1), on myeloid-derived suppressor cells (MDSCs), promoting their immunosuppressive phenotype (17, 18).

## PD-1/PD-L1 axis in cancer

Programmed cell death 1 (PD-1) receptor is expressed on different immune cells, including tumor-infiltrating lymphocytes (TILs), and binds to PD-L1, which is expressed on myeloid regulatory cells and nonimmune cells such as malignant cells (Figure 1). Inducible expression of PD-L1 at the tumor site is involved in cancer escape because its binding to PD-1 on effector T cells causes T cell exhaustion and apoptosis, while apoptosis of regulatory T cells is inhibited (19). PD-L1 expression has been correlated with poor clinical outcomes, and PD-1/PD-L1–targeted inhibitors can lead to effective clinical responses in some patients with different types of cancer. Combination therapies based on PD-1/PD-L1 blockade for a wide range of cancer types have been introduced and can improve patient survival (20). One approach uses HIF inhibitors in combination with anti–PD-1/PD-L1 therapy.

## PD-L1/HIF cross-talk and therapeutic applicability

Importantly, HIF-1α inhibition synergizes with anti–PD-1 therapy to inhibit tumor development (Figure 1). For instance, in vivo treatment with PX-478 and anti–PD-1 antibodies suppresses tumor growth and prolongs animal survival, which is associated with downregulation of EMT phenotypes, lower immunosuppression, and enhanced TIL homing (21). In a murine melanoma model, inhibition of HIF-1α transcriptional activity results in CCL2- and CCL5-mediated increases in NK cells and CTLs in the tumor bed, and improves the antitumor potential of peptide vaccination and anti–PD-1 blocking antibody (22).

In this issue of the *JCI*, Salman et al. report on the development of a low-molecular-weight agent that inhibited HIF-1/2–mediated gene expression in tumor cells and suppressed tumor growth (23). The synthesized low-molecular-weight agent targeted both HIF-1 and HIF-2, inducing HIF-α subunit degradation and thus inhibiting activation of HIF-1– and HIF-2–target gene transcription. Genes affected by this inhibition encoded regulators of angiogenesis, glycolysis, and immunity. Administration of the HIF inhibitor in tumor-bearing mice resulted in inhibition of tumor growth. The smaller tumors were associated with lower tumor vascularization due to decreased expression of multiple genes encoding angiogenic factors, and lower immunosuppression due to diminished expression of genes encoding proteins mediating immunosuppression. Key metabolic and signal-transduction pathways driving cancer progression were also blocked. In vivo alterations of the tumor immune environment involved fewer protumorigenic tumor-associated macrophages (TAMs) and MDSCs due to the suppression of IL-4, IL-13, and CXCL1 expression, and elevated infiltration by antitumor CTLs and NK cells attracted by elevated CXCL9 and CXCL10 levels. Importantly, the HIF inhibitor boosted the percentage of mice with a complete response to anti–PD-1 CPI from 25% to 67% (23).

Also in this issue of the *JCI*, Bailey, Liu, et al. demonstrate that pharmaceutical or genetic targeting of HIF-1α abrogated PD-L1–mediated immune evasion by suppressing PD-L1 expression on malignant cells and myeloid regulatory cells (MDSCs and TAMs), causing reactivation of TILs and tumor rejection (24). HIF-1α inhibition in vivo downregulated PD-1 expression, upregulated cytolytic effector molecules granzyme B and perforin, and decreased apoptosis in TILs in the tumor microenvironment. Importantly, HIF-1α inhibition potentiated immunotherapeutic effects of anti–CTLA-4 therapy in experimental models. The authors also reported an interesting observation; although HIF-1α inhibition blocked PD-L1 expression at the tumor site, it induced PD-L1 in normal tissues. This finding is important since high incidences of immune-related adverse events associated with CPI therapy are well documented. Thus, it is conceivable that HIF-1α inhibition represents a promising companion for different CPIs. Promotion of immune tolerance by differential regulation of PD-L1 in normal and cancer tissues is an important approach for safer and more successful immunotherapy (24).

## Conclusions

PD-L1 expression is controlled by several signaling pathways. Recent data indicate that hypoxia is also able to upregulate expression of PD-L1 in malignant cells via HIF-1α and HIF-2α (17). For instance, analysis of the melanoma genome atlas data shows a correlation of the HIF-1 signaling signature with PD-L1 mRNA expression, especially with IFN-γ–induced expression of PD-L1 mRNA (25). Bailey, Liu, et al. confirmed these results (24). The findings on differential regulation of PD-L1 expression by HIF suggest that a combination of HIF inhibition with some CPIs, like anti–CTLA-4, for example, should be experimentally verified. Salman et al. also support the notion that highly specific and nontoxic HIF inhibitors deserve further investigation when applied alone or in combination with CPIs and other targeted therapy (23). Testing these approaches in different tumor models will be crucial to developing effective cancer treatments. Understanding the potential synergistic effects of HIF-1 on cells in the cancer milieu with other tumor-associated factors like TGF-β, IL-1, hormones, and growth factors is another important direction for multitargeted approaches and for development of safe biological therapies. Studies must also reveal strategies that elaborate the effect of HIF inhibitors on cancer stem cell progenitors, intratumoral endothelial cells, and cancer-associated fibroblasts in conjunction with immune effectors and regulators to control the tumor metastatic potential and chemo- and radioresistance in different models.

## Version 1 05/02/2022

Electronic publication

## References

[1] Sebestyén A (2021). Hypoxia signaling in cancer: from basics to clinical practice. Pathol Oncol Res.

[2] Elzakra N, Kim Y (2021). HIF-1α metabolic pathways in human cancer. Adv Exp Med Biol.

[3] Semenza GL (2000). Hypoxia, clonal selection, and the role of HIF-1 in tumor progression. Crit Rev Biochem Mol Biol.

[4] Semenza GL (2002). HIF-1 and tumor progression: pathophysiology and therapeutics. Trends Mol Med.

[5] Lappano R (2022). Multifaceted interplay between hormones, growth factors and hypoxia in the tumor microenvironment. Cancers (Basel).

[6] Fallah J, Rini BI (2019). HIF inhibitors: status of current clinical development. Curr Oncol Rep.

[7] Tang W, Zhao G (2020). Small molecules targeting HIF-1α pathway for cancer therapy in recent years. Bioorg Med Chem.

[8] Karakashev SV, Reginato MJ (2015). Progress toward overcoming hypoxia-induced resistance to solid tumor therapy. Cancer Manag Res.

[9] Lee S-H (2015). A group of novel HIF-1α inhibitors, glyceollins, blocks HIF-1α synthesis and decreases its stability via inhibition of the PI3K/AKT/mTOR pathway and Hsp90 binding. J Cell Physiol.

[10] Lou JJW (2010). Inhibition of hypoxia-inducible factor-1alpha (HIF-1alpha) protein synthesis by DNA damage inducing agents. PLoS One.

[11] Koh MY (2008). Molecular mechanisms for the activity of PX-478, an antitumor inhibitor of the hypoxia-inducible factor-1alpha. Mol Cancer Ther.

[12] Lee K (2009). Anthracycline chemotherapy inhibits HIF-1 transcriptional activity and tumor-induced mobilization of circulating angiogenic cells. Proc Natl Acad Sci U S A.

[13] Li L (2006). Hypoxia-inducible factor-1 inhibition in combination with temozolomide treatment exhibits robust antitumor efficacy in vivo. Clin Cancer Res.

[14] Jasinghe VJ (2008). ABT-869, a multi-targeted tyrosine kinase inhibitor, in combination with rapamycin is effective for subcutaneous hepatocellular carcinoma xenograft. J Hepatol.

[15] Viry E (2014). Autophagic degradation of GZMB/granzyme B: a new mechanism of hypoxic tumor cell escape from natural killer cell-mediated lysis. Autophagy.

[16] Berchem G (2016). Hypoxic tumor-derived microvesicles negatively regulate NK cell function by a mechanism involving TGF-β and miR23a transfer. Oncoimmunology.

[17] Noman MZ (2014). PD-L1 is a novel direct target of HIF-1α, and its blockade under hypoxia enhanced MDSC-mediated T cell activation. J Exp Med.

[18] Deng J (2019). Hypoxia-induced VISTA promotes the suppressive function of myeloid-derived suppressor cells in the tumor microenvironment. Cancer Immunol Res.

[19] Han Y (2020). PD-1/PD-L1 pathway: current researches in cancer. Am J Cancer Res.

[20] Yi M (2022). Combination strategies with PD-1/PD-L1 blockade: current advances and future directions. Mol Cancer.

[21] Luo F (2022). HIF-1α inhibition promotes the efficacy of immune checkpoint blockade in the treatment of non-small cell lung cancer. Cancer Lett.

[22] Lequeux A (2021). Targeting HIF-1 alpha transcriptional activity drives cytotoxic immune effector cells into melanoma and improves combination immunotherapy. Oncogene.

[23] Salman S (2022). HIF inhibitor 32-134D eradicates murine hepatocellular carcinoma in combination with anti-PD1 therapy. J Clin Invest.

[24] Bailey C (2022). Targeting HIF-1α abrogates PD-L1–mediated immune evasion in tumor microenvironment but promotes tolerance in normal tissues. J Clin Invest.

[25] van Duijn A (2022). A secondary role for hypoxia and HIF1 in the regulation of (IFNgamma-induced) PD-L1 expression in melanoma. Cancer Immunol Immunother.

